# Prevalence and risk factors for depression and anxiety among outpatient migraineurs in mainland China

**DOI:** 10.1007/s10194-012-0442-9

**Published:** 2012-04-01

**Authors:** Na Yong, Hua Hu, Xiaoping Fan, Xuelian Li, Li Ran, Yuan Qu, Yunfeng Wang, Ge Tan, Lixue Chen, Jiying Zhou

**Affiliations:** 1 Department of Psychiatry, The First Affiliated Hospital of Chongqing Medical University, No. 1, Youyi Road, Chongqing, 400016 China; 2 Department of Neurology, The First Affiliated Hospital of Chongqing Medical University, No. 1, Youyi Road, Chongqing, 400016 China; 3 Laboratory Research Center, Chongqing Key Laboratory of Neurology, The First Affiliated Hospital of Chongqing Medical University, Chongqing, 400016 People’s Republic of China

**Keywords:** Anxiety, Cross-sectional study, Depression, Migraine, Risk factor

## Abstract

This study aimed to investigate the prevalence and risk factors for anxiety and depression symptoms in outpatient migraineurs in mainland China. In addition, we evaluated whether the Hospital Anxiety and Depression Scale (HADS) provided sufficient validity to screen depression and anxiety. A cross-sectional study was conducted consecutively at our headache clinic. Migraine was diagnosed according to International Classification of Headache Disorders, 2nd edition (ICHD-II). Demographic characteristics and clinical features were collected by headache questionnaire. Anxiety and depression symptoms about migraineurs were assessed using HADS. Several questionnaires were simultaneously used to evaluate patients with depressive disorder including the Hamilton Depression Rating Scale-17 (HAMD), Hamilton Anxiety Rating Scale (HAMA) and HADS. Pearson correlation analysis was applied to test the validity of HADS. 176 outpatients with migraine (81.8 % female) were included. Overall, 17.6 and 38.1 % participants had depression and anxiety, respectively. Possible risk factors for depression in migraineurs included headache intensity of first onset of migraine, migraine with presymptom, migraine with family history and migraine disability. The possible risk factors for anxiety included fixed attack time of headache in one day and poor sleeping, and age represented a protective factor for anxiety. The correlation coefficient of HADS-A and HADS-D with HAMA and HAMD was 0.666 and 0.508, respectively (*P* < 0.01). This study demonstrates that depression and anxiety comorbidity in our mainland Chinese migraineurs are also common, and several risk factors were identified that may provide predictive value. These findings can help clinicians to identify and treat anxiety and depression in order to improve migraine management.

## Introduction

Migraine and depression are highly prevalent, and both represent disabling diseases in populations. Studies have found that the prevalence of migraine was 17.1 % in females and 5.6 % in males [1]. In addition, epidemiological studies found an association between migraine and depression [2–5], indicating that the lifetime prevalence of depression in migraine ranged from 18.8 to 42 % [2, 3, 6]. Interestingly, migraineurs were 2.2–4.0 times more likely to suffer depression than non-migraineurs [7], and there was an increasing suicidal tendency and attempts in migraine with comorbid depression [8]. Breslau concluded that association between migraine and major depression may result from bi-directional influences; each disorder would increase the risk for first onset of the other [9].

Previous studies have also found that anxiety, particularly general anxiety disorder, was common in migraineurs [4]. Furthermore, a study using Chinese subjects from Taiwan found that migraine were associated with both depression and anxiety [10] and the onset of anxiety typically preceding the onset of migraine with the risk of depression increasing subsequently [11]. However, due to economic and cultural differences, the pattern in mainland China may be different than that in Western countries or Taiwan**.**

Several studies supported that psychiatric comorbidities could promote the episodic headaches transform into chronic syndromes [12, 13]. This transformation may increase the difficulty in both treatment and headache-related disability [2]. Therefore, these findings indicate that psychiatric comorbidity must be treated in timely manner; however, the identification and diagnosis of psychiatric comorbidity in migraineurs is much lower, especially in China. Understanding the risk factors associated with depression and anxiety in migraineurs will provide helpful information for clinicians to prevent and treat these disorders. To date, only a few studies have examined the demographics and clinical features in patients with headaches associated with depression [14, 15], and no studies have investigated the risk factors for depression and anxiety in migraineurs**.**

The Hospital Anxiety and Depression Scale (HADS) is a fast screening tool, to screen depression and anxiety symptoms in somatic, psychiatric and primary care patients [16].

Therefore, the purpose of our study was to assess the prevalence and risk factors for anxiety and depression in migraine outpatients in mainland China. In addition, we also tested the validity of the HADS to determine whether this tool displays sufficient validity to screen depression and anxiety symptoms in migraineurs.

## Methods

### Subjects and procedures

One hundred and eighty-five consecutive migraine patients were included in this study. These patients visited the headache outpatient clinic in the First Affiliated Hospital of Chongqing Medical University in China from September 2009 to January 2011. Migraine diagnosis was conducted by a neurologist according to the ICHD-II [17], and the diagnosis was confirmed by a headache specialist. The following criteria were also utilized. The course of migraine must be more than 1 year. Subjects were excluded if they had experienced significant life-changing events (e.g. seeing others being killed, death and serious injury; hearing some terrible things; being raped; being divorced, etc.) recently [18], or were prescribed antidepressants or other psychotropic drugs in the past 2 weeks. According to these criteria, nine patients were excluded from the study, and 176 people included in the final study. 94.3 % of the subjects were of Chinese Han ethnicity.

To test the validity of the HADS, 30 consecutive major depression disorder patients (66.7 % female) were included who visited the psychiatry department in the same hospital from January 2011 to May 2011. The age of the subjects ranged from 20 to 59 years (average 36.5 ± 10.9 years). Depression was diagnosed by a psychiatry specialist according to the Structured Clinical Interview provided in the DSM-IV-text revision (TR) Axis I Disorders [18]. The patients were also assessed by a psychiatrist. 98 % of the subjects were of Chinese Han ethnicity.

The study protocol was approved by the Ethical Committee at Chongqing Medical University and in accordance with the Declaration of Helsinki. All patients had given written consent to the study.

All headache information was collected by three neurologists through face-to-face interviews. The findings were then reviewed by a headache specialist, and anxiety and depression symptoms of the migraineurs were assessed by a psychiatrist. Demographic characteristics and clinical features were collected using a headache questionnaire. Demographic characteristics included age, sex, employment status, educational level (four categories: ≤6, 7–9, 10–12, >12 years), dwelling (three categories: living in town, in suburban area, in countryside) etc.; and clinical features of the migraine headache included the first onset age, headache pain intensity, headache nature, locus, duration, presymptom (e.g., fluctuating mood, drowsiness, reduced activity, yawning, desire to eat, neck tightness, etc.), the average monthly headache frequency in the recent 3 months, family migraine history (first-degree relatives), migraine disability, sleeping (i.e., excellent, better, bad, worst), etc. Finally, a question about life events (Were there any events which were difficult to handle that influenced your mood in the last month?) was included as well as the psychiatry medication history in the past 2 weeks.

### Measures

Headache pain intensity was assessed by an 11-point pain scale (0–10) with 0 indicating “no headache” and 10 indicating “headache as severe as I can imagine” [19]. 1–3 was defined as “mild”; 4–6 as “moderate”; and 7–10 as “severe”. Migraine-related disability was assessed by The Migraine Disability Assessment questionnaire (MIDAS) [20]. Participants were divided into four grades by MIDAS using previously validated scores based on lost-time due to headaches (i.e., minimal, mild, moderate, and severe disability). In addition, ICHD-II was also applied to divide the patients into subgroups, and the subgroups included migraine with aura (MA) and migraine without aura (MOA) or episodic migraine (EM) and chronic migraine (CM) [17].

Anxiety and depression were assessed utilizing HADS [16]. It is a well-established self-rating instrument with 14 items, and incorporates seven anxiety questions (HADS-A) and seven depression questions (HADS-D). Each item is scored from 0 (not present) to 3 (maximally present), representing a 4-point scale. Because the total score of each subscale is 21, the total score of each subscale that is greater than eight indicates that the person may have anxiety (ANX) or depression (DEP) symptoms. If the total score of HADS-D ≥8 and HADS-A <8, the person has pure depressive symptoms (pure DEP). If the total score of HADS-A ≥8 and HADS-D <8, the person has pure anxiety symptoms (pure ANX). When the total score of both HADS-A and HADS-D ≥8, it accounts for that the person has comorbid symptoms (COM). Previous studies indicated that the correlation of HADS-D and HADS-A with other commonly used anxiety and depression questionnaires such as Beck Depression Inventory(BDI), State-Trait Anxiety Inventory (STAI), and SCL-90 Anxiety and Depression subscales, respectively, were between 0.60 and 0.80, the validity and reliability of HADS are good [21, 22].

In order to check the validity of HADS in Chinese subjects, we simultaneously evaluated major depression disorder patients using the Hamilton Depression Rating Scale-17 (HAMD-17), Hamilton Anxiety Rating Scale (HAMA), and HADS. Previous studies have found that HAMD and HAMA also have good valid and reliability [23–25].

### Statistical analysis

All data were analyzed using the SPSS 17.0 statistics package. Continuous variables data were expressed as mean ± SD. One-way ANOVA and *χ* ^2^ tests were used for comparison when appropriate. We used binary logistic regression to calculate the odds ratios (ORs) for pure DEP, pure ANX and COM in persons having EM and CM, and also analyzed both genders. We evaluated potential confounding by adjusting for age and education, and investigated potential interaction effects between gender and pure DEP, pure ANX and COM by including the product of the two variables in the logistic regression analyses. We used the Wald *χ* ^2^ statistic to test the interaction coefficients.

When analyzing the risk factors for depression or anxiety in migraineurs, we used binary logistic regression with a backward progression. The dependent variable was the presence or absence of depression or anxiety symptoms. The 18 independent variables consisted of the following: age (three categories: <30, 30–45, >45 years), gender, educational years, profession, dwelling, the first-onset age of migraine (<20, 20–35, >35 years), headache pain intensity of first onset of migraine (none, mild, moderate, severe), average monthly headache frequency in the recent 3 months (≤8, 9–14, ≥15 days per month), frequency of headache in 1 year (<1, 2–3, 4–6, >6 months), the most painful degree (none, mild, moderate, severe), fixed attack time in one day (yes/no), fixed attack time on year (yes/no), presymptom, course of disease (≤15, 16–30, >30 years), sleeping, family history of migraine (yes/no), migraine disability (minimal, mild, moderate, severe), and headache pain intensity of migraine now (none, mild, moderate, severe).

When checking the validity of the HADS, the normality of data was first tested for HAMD, HAMA, and HADS, and the results indicated that all demonstrated normal distributions. We then used the Pearson product-moment correlation analysis to analyze the correlation of HADS-A and HADS-D with HAMA and HAMD.

A significance level of *P* < 0.05 was chosen in all statistical hypotheses, and all statistical tests were two-sided.

## Results

### Migraine characteristics

The migraine samples including 144 (81.8 %) females and 32 (18.2 %) males, ranging in age from 14 to 63 years (average 39.1 ± 11.6 years). The average first-onset age of migraine was 23.8 ± 10.3 years. The average course of migraine was 15.4 ± 11.0 years. The headache frequency during the last 3 months were 106 patients (60.2 %) with ≤8 days/month, 17 (9.7 %) with 9–14 days/month, and 53 patients (30.1 %) with ≥15 days/month. A significant difference was observed for depression and anxiety in different frequency of headache (DEP: *F* = 3.11, *P* = 0.047; ANX: *F* = 3.48, *P* = 0.033). Our results indicated that the seriousness of depression and anxiety symptoms were correlated with the frequency of the headache. The average intensity score of the headache in a recent month was 7.4 ± 1.3. The migraine subtypes are shown in Table 1.

**Table 1:** The subtypes of migraine and the depression and anxiety symptoms in migraineurs, *N* (%)

	*N* (%)	MOA	MA	EM	CM	No mental disorder	Pure DEP	Pure ANX	COM
Total	176 (100)	152 (86.4)	24 (13.6)	123 (69.9)	53 (30.1)	104 (59.1)	5 (2.8)	41 (23.3)	26 (14.8)
Male	32 (18.2)	24 (75)	8 (25)	22 (68.8)	10 (31.3)	24 (75.0)	0 (0)	5 (15.6)	3 (9.4)
Female	144 (81.8)	128 (88.9)	16 (11.1)	101 (70.1)	43 (29.9)	80 (55.6)	5 (3.5)	36 (25.0)	23 (16.0)

*MOA* migraine without aura, *MA* migraine with aura, *EM* episode migraine, *CM* chronic migraine, *DEP* depressive symptoms, *ANX* anxiety symptoms, *COM* comorbid depressive and anxiety symptoms

### Depression symptoms and anxiety symptoms in migraineurs

The average depression score of migraineurs was 4.4 ± 3.6, and depression symptoms (DEP) occurred in 17.6 % (31 of 176). The prevalence rate of depression symptoms in females was twice than that in males (19.4 vs. 9.4 %), but no significant difference was found between sex (*P* > 0.05). The prevalence rate of depression symptoms in MOA was 17.1 % (26 of 152), in MA was 20.8 % (5 of 24); and 13.8 % (17 of 123) in EM, 26.4 % (14 of 53) in CM. A significant difference of prevalence rate was detected in depression symptoms between EM and CM (*χ* ^2^ = 4.05, *P* = 0.044).

The average anxiety score of migraineurs was 6.5 ± 3.8. Anxiety symptoms (ANX) occurred in 38.1 % (67 of 176). The prevalence rate of anxiety symptoms in females was higher than in males (41.9 vs. 25.0 %), but no significant difference was observed between sex (*P* > 0.05). The prevalence rate of anxiety symptoms in MOA was 37.5 % (57 of 152), in MA was 41.7 % (10 of 24); and 33.3 % (41 of 123) in EM, 49.1 % (26 of 53) in CM, there was significant difference between EM and CM (*χ* ^2^ = 3.88, *P* = 0.049).

In all migraine patients there were only 2.8 % comorbid pure DEP, 23.3 % comorbid pure ANX, and 14.8 % comorbid both DEP and ANX. Depression and anxiety symptoms were more common in females (Table 1).The odds ratio of pure DEP, pure ANX and COM prevalence in persons with CM compared to EM are shown in Table 2. COM was more likely to occur in CM, especially in females having CM rather than in those with EM. No significant associations were found between pure DEP and pure ANX in either the CM or EM groups.

**Table 2:** The odds ratio of pure depression symptoms, pure anxiety symptoms and comorbid symptoms prevalence in patients with CM compared with EM

Variance	HADS	*n*	*P*	OR	95 % CI
CM		53			
Both genders	No disorder	24		1	
	Pure DEP	3	0.068	6.39	0.87, 46.99
	Pure ANX	15	0.055	2.34	0.98, 5.57
	COM	11	0.011	3.72	1.35, 10.28
Male	No disorder	6		1	
	Pure DEP	0			
	Pure ANX	2	0.692	0.45	0.01, 23.53
	COM	2	0.743	2.02	0.03, 133.74
Female	No disorder	18		1	
	Pure DEP	3	0.070	6.23	0.86, 44.90
	Pure ANX	13	0.091	2.21	0.88, 5.51
	COM	9	0.049	2.95	1.00, 8.68

All numbers were adjusted for age, and education. Gender interaction was tested: gender × pure DEP, *P* = 0.091; gender × pure ANX, *P* = 0.663; gender × COM, *P* = 0.234

*DEP* depressive symptoms, *ANX* anxiety symptoms, *COM* comorbid depressive and anxiety symptoms

### Risk factors for depression in migraineurs

Logistic regression analysis revealed that the following four factors were associated with depression in migraineurs: (1) headache intensity of first onset of migraine, (2) presymptom of migraine (mainly including: fluctuating mood, drowsiness, reduced activity, yawning), (3) migraine family history, and (4) migraine disability. We found the moderate headache intensity was the highest risk factor for depression as compared to mild and severe headache intensity in first onset of migraine. Presymptom and family migraine history were also risk factors for depression; and the mild, moderate and severe disability were all significantly different than minimal disability on the outcome measure for depression (Table 3).

**Table 3:** Risk factors for depression in migraineurs, analyzed by Binary backward logistic regression

Variance	*P*	OR	95.0 % CI
Headache intensity of first onset			
Mild		1	
Moderate	0.037	4.86	1.10, 21.56
Severe	0.248	2.34	0.49, 11.13
Presymptom of migraine			
No		1	
Yes	0.007	7.78	1.75, 34.63
Family migraine history			
No		1	
Yes	0.017	3.38	1.25, 9.14
Migraine disability			
Minimal		1	
Mild	0.039	3.60	1.07, 12.15
Moderate	0.014	5.03	1.39, 18.25
Severe	0.014	5.12	1.40, 18.79

### Risk factors for anxiety in migraineurs

Logistic regression analysis revealed that age was a protective factor for anxiety. For example, a lower incidence of anxiety was correlated with the increasing age of the patient. However, fixed attack time of headache in one day and poor sleeping were the risk factors associated with anxiety in migraineurs (Table 4).

**Table 4:** Risk factors for anxiety in migraineurs, analyzed by binary backward logistic regression

Variance	*P*	OR	95.0 % CI
Age			
<30 years		1	
30–45 years	0.003	0.19	0.06, 0.56
>45 years	0.018	0.25	0.08, 0.79
Fixed attack time of headache in one day			
No		1	
Yes	0.016	4.26	1.30, 13.94
Sleeping			
Excellent		1	
Better	0.297	2.52	0.44, 14.26
Bad	0.004	13.46	2.30, 78.87
Worst	0.003	36.34	3.38, 390.99

### Validity of HADS

Correlation analysis was used to analyze the results of HAMD, HAMA and HADS from the patients of major depression disorder, and the results of the correlation coefficient of HADS-A with HAMA was 0.666 and HADS-D with HAMD was 0.508 (Table 5). Of these 30 major depressive disorder patients 27 patients had the total score of HADS-D ≥8, so the sensitivity of HADS-D was 90 % (27/30); and there were 26 patients with a total score of HADS-A ≥8, so the sensitivity of HADS-A was 86.7 % (26/30).

**Table 5:** Pearson correlation coefficients of HADS-D and HADS-A with HAMD and HAMA

	HAMA	HAMD	HADS-A	HADS-D
HADS-A	0.666^a^	0.506^a^	1.000	0.631^a^
HADS-D	0.581^a^	0.508^a^	0.631^a^	1.000

*HADS-A* Hospital Anxiety and Depression Scale (anxiety subscale), *HADS-D* Hospital Anxiety and Depression Scale (depression subscale), *HAMA* Hamilton anxiety rating scale, *HAMD* Hamilton depression rating scale-17

^a^Correlation is significant at the 0.01 level (2-tailed)

## Discussion

### Incidence of depression and anxiety symptoms

In our headache outpatient clinic, we used HADS to detect depression and anxiety symptoms in migraine patients. The results found that the incidence of depression symptoms was 17.6 %. This finding was lower than the rates observed in other clinical studies, including 20 % seen in UK [26] and 23.1 % seen in Italy [27], and are also lower than the rate in a population-based study which ranged from 28.5 to 42 % in USA [4, 5]. Wang [10] investigated the prevalence of depression in the elderly headache patients in Taiwan, and found that the prevalence of depression was 13.4 %; however, headache subtypes were not examined. The incidence of anxiety symptoms was 38.1 % in our samples, which was similar to the results of a study from Italy (38.1 %) [27], but lower than a population-based study (51 %) from the USA [28].

These differences may result from inherent differences associated with race and culture [29] or different evaluation instruments. Chinese culture tends to express depression somatically (e.g., fatigue, sleep problems, headache, muscle pain, etc.), but tends to deny psychological symptoms, especially depression. Therefore, depression has lower rate of detection and diagnosis, and is easily misdiagnosed as neurasthenia [30, 31]. In our study, when we informed patients that we would assess their depression and anxiety status, some patients felt irritated and denied that they had a psychiatric disorder, and they tried to refuse the assessment. However, when investigators explained that it was just a mood assessment and told them that the correct assessment could help to treat their headache, the patients agreed to comply. When they were completing the questionnaire, they seemed to avoid of expressing their real depressive mood.

The seven depression items of HADS mainly assess the mood symptoms including lowered mood, anhedonia, and psychomotor retardation; however, HADS lacks assessment items regarding somatic symptoms. In previous studies, DSM-IV, HAMD, HAMA, or Beck Depression Inventory (BDI) were used to evaluate mood, which contain items about somatic symptoms [2, 3]. Therefore, the use of HADS in our study to a certain extent may have led to our lower detection rate of depression.

### The validity of HADS

Previous studies indicated that HADS is a valid and reliable instrument to screen for depression and anxiety symptoms [21]. Furthermore, Juang [22] found that HADS was a valid and reliable in a study of headache patients in Taiwan. Because this test requires less time to complete, we used HADS to evaluated depression and anxiety symptoms in migraineurs. As our rate of depression or anxiety symptoms was lower than previous studies, we questioned whether HADS represented a valid measure. Therefore, we compared the findings from HADS to that of HAMD and HAMA in patients with major depression disorder. The correlation coefficient of HADS-A and HADS-D with HAMA and HAMD were 0.666 and 0.508, respectively, similar to previous study [21]. But the correlation of HADS-D with HAMD was somewhat lower, although the sensitivity of HADS-D was good; this might be the cause that led to lower detection results in our study. Therefore, our findings suggest that future studies could utilize BDI and Beck Anxiety Inventory (BAI) or HAMD and HAMA to evaluate depression or anxiety symptoms in migraineurs. These scales represent professional assessment scales that have a better validity and reliability [24, 25, 32].

### The relationship of the DEP and ANX with migraine characteristics and subgroups

Our study demonstrates that migraine was associated with depression and anxiety, and the prevalence was higher in females than males, which is consistent with the previous studies [2, 3]. Moreover, the prevalence of depression and anxiety in the general population was also higher in females than in males [33]. We observed that frequency of headaches was associated with more serious depression and anxiety symptoms in agreement with Mitsikostas’ results [34]. We also found a stronger relationship of COM with CM than with EM. These results indicate that doctors should check whether the patient has comorbid depression or anxiety symptoms if headaches occur frequently in a migraineur, especially to be vigilant in managing the COM in CM.

### Risk factors for DEP and ANX in patients with migraine

Fewer studies explored the risk factors for mental disorder in migraineurs. In this study, we found that only moderate headache among the first onset of migraine was associated with depression. This result was contrary to those of Mitsikostas’ study [34] where the frequency and the duration of the headache attacks were significantly associated with depression or anxiety, not the intensity of headache. However, he did not divide the headache into subtypes, and the degree of headache with first onset of migraine may be confounded by recall bias in our study, potentially leading to this discrepancy.

We also found that headache-related disability was a risk factor for depression consistent with Jelinski’s study [14], who observed that severe headache-related disability was independently associated with depression. However, he did not divide the headache into subtypes either. In addition, we discovered another two risk factors for depression including the family migraine history (first-degree relatives) and presymptoms of migraine (i.e., fluctuating mood, drowsiness, reduced activity, yawning). Merikangas [35] found a significant association between migraine and depression in both probands as well as the relatives. In our study, we observed that patients were more likely to suffer from depression if the patients had a family history of migraines.

We also found that the perception of presymptoms of migraine enabled patients to forecast headache attacks in advance, a finding that has not yet been reported in previous studies. This perception may induce a patient’s fear of headache and depression symptoms. In this finding also exists a confounding factor, fluctuating mood, which is one of the presymptoms of migraine that might be assessed in the outcome measure and hence be a possible confounder. Even so, clinicians should note depression symptoms if migraine patients present presymptoms for a better and timely treatment.

We only found two factors that were independent risk factors for anxiety including a fixed attack time of headache in one day and poor sleeping. Frisoni’s study [36] indicated that anxiety was independently associated with quality of sleep. In addition, Morrison [37] investigated sleep problems in 943 adolescents from the general population, and found that adolescents reporting sleep problems were more anxious and depressed. To date, no studies have identified a relationship between fixed attack time and mood disorders, which is potentially related to the fluctuations of ovarian hormones [38]. Interestingly, we observed that age was a protective factor for anxiety. Only one investigation reported a decrease of depression disorder prevalence in older age groups [2].

### The necessity of treatment for mental disorder

Migraine and depression significantly decrease the quality of life [2] and lead to a worse prognosis, chronic disease, and a decreased response to treatment [39]. Pesa [40] indicated that migraine comorbid with depression or anxiety also results in significantly higher medical costs as compared to migraine alone. Therefore, early diagnosis and treatment for psychiatric comorbidity are very important. If comorbid depression is not recognized and treated effectively, successful headache management is unlikely. Furthermore, it is obviously not appropriate for doctors to exclusively focus on the treatment of headache symptoms of migraine patients and neglect the psychiatric comorbidity under the traditional medical model. When treating migraine headaches, clinicians should be alert to psychiatric comorbidity, and then treat psychiatric comorbidity in a timely manner in order to prevent the functional lesion of migraineurs. This strategy would certainly be beneficial to the improvement of their life quality.

### Limitations

Our study has some methodological limitations. First, migraine patients in our study were limited to those who did not have great life events recently and those who had not taken antidepressants or other psychotropic drugs in the past 2 weeks. The purpose of these exclusion criteria was to prevent confounders that may affect the mood characteristics. Second, the sample size was not large enough to detect a difference between MA and MOA. Third, we checked the validity of HADS only in major depression disorder patients in our psychiatric department, but not in migraine patients. We believed that the results would be more accurate if the validity of the HADS was evaluated among patients that were exactly diagnosed with major depression disorder.

This study has several clinical implications. First, the comorbidity of depression and anxiety in mainland Chinese migraineurs is common. When clinicians treat headache of migraine, they should pay close attention to psychiatric comorbidity, and treat these disorders in a timely manner. Second, as our results suggest, multiple risk factors are associated with depression and anxiety in migraineurs. Thus, when migraineurs come to seek help from clinician, clinicians should pay attention to these factors because these risk factors can be helpful for predicting and identifying mood symptoms.

## Abbreviations

HADS: Hospital Anxiety and Depression Scale
ICHD-II: International Classification of Headache Disorders, 2nd edition
MIDAS: The migraine disability assessment questionnaire
MA: Migraine with aura
MOA: Migraine without aura
EM: Episodic migraine
CM: Chronic migraine
ANX: Anxiety symptoms
DEP: Depression symptoms
HADS-A: Hospital Anxiety and Depression Scale (anxiety subscale)
HADS-D: Hospital Anxiety and Depression Scale (depression subscale)
HAMD: Hamilton Depression Rating Scale
HAMA: Hamilton Anxiety Rating Scale
